# Rare Freshwater Ciliate *Paramecium chlorelligerum* Kahl, 1935 and Its Macronuclear Symbiotic Bacterium “*Candidatus* Holospora parva”

**DOI:** 10.1371/journal.pone.0167928

**Published:** 2016-12-16

**Authors:** Olivia Lanzoni, Sergei I. Fokin, Natalia Lebedeva, Alexandra Migunova, Giulio Petroni, Alexey Potekhin

**Affiliations:** 1 Department of Biology, University of Pisa, Pisa, Italy; 2 Department of Invertebrate Zoology, Faculty of Biology, Saint Petersburg State University, Saint Petersburg, Russia; 3 Centre of Core Facilities “Culture Collections of Microorganisms”, Research Park, Saint Petersburg State University, Saint Petersburg, Russia; 4 Department of Microbiology, Faculty of Biology, Saint Petersburg State University, Saint Petersburg, Russia; University of Minnesota, UNITED STATES

## Abstract

Ciliated protists often form symbioses with many diverse microorganisms. In particular, symbiotic associations between ciliates and green algae, as well as between ciliates and intracellular bacteria, are rather wide-spread in nature. In this study, we describe the complex symbiotic system between a very rare ciliate, *Paramecium chlorelligerum*, unicellular algae inhabiting its cytoplasm, and novel bacteria colonizing the host macronucleus. *Paramecium chlorelligerum*, previously found only twice in Germany, was retrieved from a novel location in vicinity of St. Petersburg in Russia. Species identification was based on both classical morphological methods and analysis of the small subunit rDNA. Numerous algae occupying the cytoplasm of this ciliate were identified with ultrastructural and molecular methods as representatives of the *Meyerella* genus, which before was not considered among symbiotic algae. In the same locality at least fifteen other species of “green” ciliates were found, thus it is indeed a biodiversity hot-spot for such protists. A novel species of bacterial symbionts living in the macronucleus of *Paramecium chlorelligerum* cells was morphologically and ultrastructurally investigated in detail with the description of its life cycle and infection capabilities. The new endosymbiont was molecularly characterized following the full-cycle rRNA approach. Furthermore, phylogenetic analysis confirmed that the novel bacterium is a member of *Holospora* genus branching basally but sharing all characteristics of the genus except inducing connecting piece formation during the infected host nucleus division. We propose the name “*Candidatus* Holospora parva” for this newly described species. The described complex system raises new questions on how these microorganisms evolve and interact in symbiosis.

## Introduction

One of the most studied ciliate genus is *Paramecium* (Ciliophora, Oligohymenophorea), which comprises nineteen valid morphospecies [1]. Some of these species seem to be cosmopolitan, while other *Paramecium* species are less widely distributed or might be even considered endemic [2, 3]. After the initial work of Müller in 1786 [4], more than forty species descriptions of the *Paramecium* genus have been published [1, 2, 5–6]. Some of these species have been retrieved only once and have not been found anymore after description. This set of species has been treated as uncertain, and some have even been abolished [2]. However, among these doubtful paramecia, some have been found and redescribed as true morphospecies during last two decades: *P*. *duboscqui* [7, 8], *P*. *nephridiatum* [9, 10], *P*. *chlorelligerum* [11, 12]. As matter of fact, the first two species are currently considered to be widely distributed, instead *P*. *chlorelligerum* can be estimated as a very rare ciliate.

This “forgotten” ciliate bears unicellular symbiotic algae in its cytoplasm, and, thus, it can be considered as a “green” *Paramecium*, like well-known *P*. *bursaria*. It has been recently found and redescribed in only a particular pond of Southern Germany, in Simmelried moorland [12]. In 2014 the same ciliate has been retrieved by us also in Peterhof (St Petersburg district, Russia) in a small permanent ditch, which, by some ecological parameters, appears to be quite similar to the German one in Simmelried [12]. The newly found freshwater locality manifests very unusual high diversity and number of green ciliates and can be considered a biodiversity hot-spot for these protists. Some representatives of the Russian population of *P*. *chlorelligerum* showed bacterial infection in the macronucleus. The morphology of these endosymbionts fits quite well to the known *Holospora*-like bacteria [13]. Indeed, representatives of this alphaproteobacterial genus, and some other closely related *Rickettsiales*, have been found in seven *Paramecium* morphospecies as well as in *Frontonia salmastra* and *Frontonia leucas* [13, 14]. These intracellular bacteria are highly infectious nuclear symbionts with unique morphology and life cycle, and they have been considered to be a very promising model organism for investigations of symbiotic associations between eu- and prokaryotes [15]. Indeed, in the last years, several studies have been performed to facilitate their use as model organisms [16–19], and they have been extensively used in evolutionary ecology studies [20–22]. Up to now, the genus *Holospora* comprises ten species [13, 23], which have been described morphologically, but only for several of them molecular characterization is available [23–27].

In the present study, we combined classical morphological approaches with molecular analysis to improve our knowledge on *P*. *chlorelligerum*, an extremely rare *Paramecium* species, examining its variability, physiology and symbiotic interactions both with unicellular algae and prokaryotic microorganisms. Infectious bacteria found in the host ciliate macronucleus were described as a new species of the genus *Holospora*.

## Materials and Methods

### Host isolation, cultivation and identification

**Fig 1:**
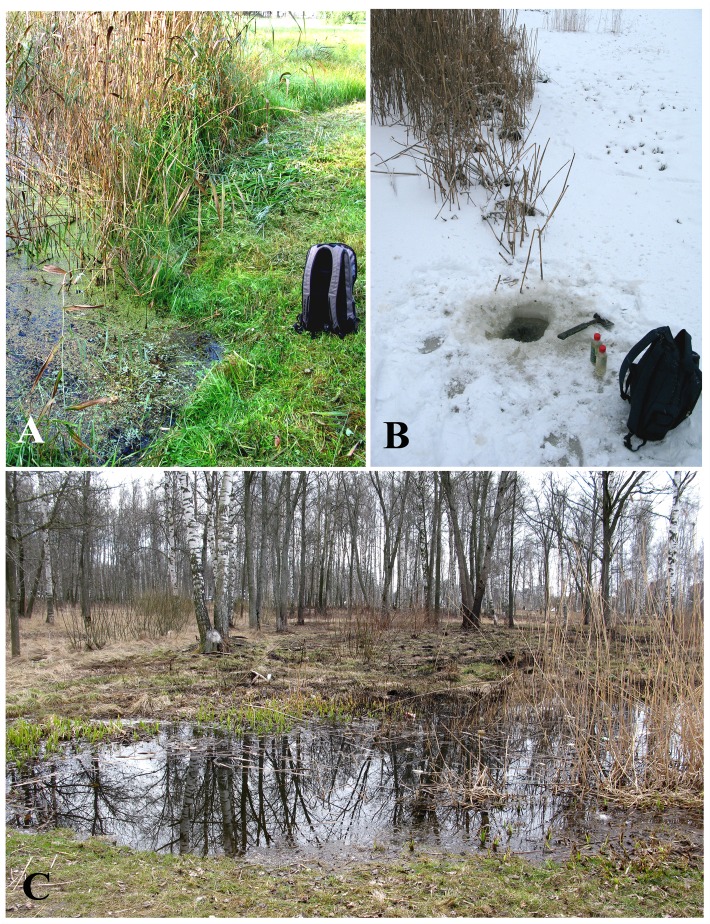
**Sampling location in Peterhof (Russia) in different seasons:** (A) August; (B) January; (C) April.

*P*. *chlorelligerum* has been found in the small but relatively deep permanent ditch in the corner of the so called English park (Peterhof, St Petersburg district, Russia (N 59°52′47″, E 29°51′56″). After the first finding, dated September 2014, sampling was repeated throughout the year covering four different seasons (Fig 1). *P*. *chlorelligerum* was constantly present among a large number of ciliated protists, many of which were inhabited by green symbiotic algae.

We succeeded to establish clonal cultures of *P*. *chlorelligerum* using combination of classical feeding medium (wheat grass medium inoculated with *Klebsiella aerogenes* or lettuce medium with the same bacterium) and addition of beta-sitosterol (Merck, Darmstadt, Germany) (0.8 mg/l) and 1/3 volume of mineral medium [28]. Cultures were kept in plastic Petri dishes inside climatic chamber Sanyo at 18°C with illumination regime of 10 hours darkness and 14 hours light by 2000 lx lamp.

*P*. *chlorelligerum* is characterized by unusually low division rate for *Paramecium* representatives (only 1–2 divisions per week). This could be related with the presence of very few food vacuoles in the cytoplasm of ciliates, indeed we could keep ciliate culture in a good condition for about one month even without feeding.

For the study, four clonal cultures established in laboratory (indexes HSG1 and HSG2 correspond to sampling of 2014 and 2015 respectively) were used: HSG1-6, HSG2-10 (without bacterial infection in the macronucleus), and infected HSG1-11 and HSG2-12. These clones are available from the Centre of Core Facilities “Culture Collections of Microorganisms”, St Petersburg State University, Russia.

Species identification of obtained clones was performed according to the ciliate morphology [11, 12], and then confirmed by molecular analysis.

### Symbiotic algae isolation, cultivation and identification

Attempts of *P*. *chlorelligerum* symbiotic algae isolation and cultivation were performed. Several paramecia cells were thoroughly washed in sterile water, then separately squashed and grinded with a glass mini-spatula on agar plates containing Bold Bristol Medium (BBM) [29], glucose (1 g/l) and 10% amino-peptide, and ampicillin (100 μg/ml) in order to minimize bacterial growth. Plates were kept at +24°C with constant daylight for four-five weeks. Several colonies of green algae were obtained directly on BBM agar plates, then grown in liquid BBM, and purified by repetitive passages on BBM plates. In the experiment, several dozens of paramecia were used, and only once cyst-like cells were observed after several weeks of slow growth. These cysts were transferred to liquid BBM, and after one week of growth a non-axenic algae culture was obtained. Identification of algae cultures was performed by phase contrast microscopy and by molecular methods (see DNA extraction and molecular characterization).

### Morphological and Ultrastructural Characterization

Living cells were immobilized for observation with the help of a special device [30]. Impregnation was performed with the silver nitrate procedure made after Champy’s fixation [31, 32], and nuclear apparatus was also stained with Feulgen method. Living and fixed cells were examined and photographed by bright field and Nomarski interference (DIC) microscopy using an Orthoplan Leitz microscope (Leitz, Germany) at ×300–1.250 magnifications with a digital camera Canon S45 and True Chrome HDII Screen, as well as with Polyvar microscope (Reichert-Jung, Austria).

For the investigation of macronuclear bacteria and cytoplasmic algal symbionts, the material was fixed for electron microscope according to a protocol routinely used [33].

### Experimental infections

Experimental bacterial infection was carried out using a homogenate prepared from infected cells according to Preer [34]. In particular, *P*. *chlorelligerum* cells were infected mixing equal volumes of a dense cell culture and of homogenate in a 3-mL depression slide, and maintained at 18°C. To check the infection development, at least ten living cells were observed 2 hours, 24 hours, 48 hours, 4 days, 7 days, and 28 days after procedure. Then infection status was controlled every 10–15 days.

### DNA extraction and molecular characterization

About 50 starved *Paramecium* cells were washed several times in sterile distilled water and fixed in 70% ethanol. Total genomic DNA was extracted using the NucleoSpin™ Plant II DNA extraction kit (Macherey-Nagel, Germany), following the protocol for mycelium.

All polymerase chain reactions (PCRs) were performed in a C1000TM Thermal Cycler (BioRad, Hercules, CA) with the TaKaRa ExTaq (TaKaRa Bio Inc., Otsu, Japan). Each PCR consisted of 35 cycles divided as follows: a preliminary denaturation step at 94°C for 3 minutes, then denaturation at 94°C for 30 seconds, annealing at variable temperature (between 50 and 55°C) for 30 seconds and elongation at 72°C for 90 seconds, and a final elongation step at 72°C for 5 minutes. In every PCR experiment, a negative control without DNA was always included. After evaluation by electrophoresis on 1% agarose gel, PCR products were purified with EuroGold CyclePure kit (EuroClone®, Milan, Italy) and directly sequenced using appropriate primers.

The amplification of the host 18S rDNA required a semi-nested PCR approach, as reported in [35], in order to achieve sufficient DNA for direct sequencing. In short, the first PCR was carried out with primers 18S F9 Euk (5’-CTGGTTGATCCTGCCAG-3’ [36]) and 18S R1513Hypo (5’-TGATCCTTCYGCAGGTTC-3’ [37]), then using as template the diluted first PCR product, a second step was performed employing two semi-nested PCRs: 1—using primers 18S F9 and Penic R1280 (5’-CGACACGTCCTAACAAGA-3’ [38]) and 2—with primers Penic F82 (5’-GAAACTGCGAATGGCTC-3’ [39]) and 18S R1513Hypo. The PCR products were then directly sequenced using eukaryotic universal internal primers, as shown in [40].

The complete ITS1-5.8S-ITS2 sequence of the ciliate was obtained following Boscaro et al. [41] and the host cytochrome *c* oxidase subunit I (COI) gene was amplified and sequenced as described by Strüder-Kypke et al. [42].

The 16S rRNA gene of *Holospora* sp. was obtained using primer pair 16S alfa F19a (5’-CCTGGCTCAGAACGAACG-3’ [43]) and R1492 (5’-GGNWACCTTGTTACGACTT-3’, modified from [44]). The almost complete 16S rDNA gene sequence was directly sequenced using primers 16S F114HoloCaedi (5’-TGAGTAACGCGTGGGAATC-3’ [23]), 16S R515ND (5’-ACCGCGGCTGCTGGCAC-3’), and 16S F785ND (5’-GGATTAGATACCCTGGTA-3’ [43]).

The amplification of 18S rDNA sequences of the algal endosymbiont and cultivated algae was performed using primers Chlo F59 (5’-CATGTCTAAGTATAAACTGCT-3’ [This study]) and Chlo R1052 (5’-CCTGACAAGGCAACCC-3’ [This study]) and directly sequenced with internal primers Chlo F194a (5’-TATTAGATAAAAGGCCGACC-3’ [This study]), Chlo R426 (5’-CTCATTCCAATTACCAGAC-3’ [This study]), Chlo F770 (5’-TGGGGGCTCGAAGAC-3’ [This study]).

### Probe design and fluorescence *in situ* hybridization

The obtained 16S rRNA gene sequences were used to design a specific probe HoloParv_645 5’-CCGTACTCTAGTCTCCC-3’ (Tm = 55.2°C). The probe was synthesized and labeled with Cy3 by Eurofins GMBH (Ebersberg, Germany), and its specificity was tested *in silico* both on Ribosomal Database Project (RDP [45]) and on TestProbe tool 3.0 (SILVA rRNA database project [46]) allowing 0 and 1 mismatches (Table 1). The probe sequence was deposited in Probebase [47].

**Table 1:** *In silico* matching of the species-specific probe HoloParv_645 against bacterial 16S rRNA gene sequences available from RDP (release 11, update 4) and SILVA (release 123) databases. The number of sequences (“hits”) which hybridize with the designed probe are reported for 0 and 1 mismatches.

Species-specific probe	RDP		SILVA	
	0 mismatches	1 mismatches	0 mismatches	1 mismatches
HoloParv_645	32 hits	1607 hits	7 hits	326 hits

Fluorescence *in situ* hybridization (FISH) experiments were carried out using different formamide concentrations in the hybridization buffer (0, 10, 20 and 30% v/v), in order to test the different stringency levels. The signal intensity was the best at 0% formamide. All FISH experiments were then performed following the protocol by Manz et al. [48] and negative controls, namely experiments without the use of probes, were always included. An additional probe, EUB338 5’-GCTGCCTCCCGTAGGAGT-3’ [49], was used as a control to exclude the presence of other intracellular bacteria and to detect unambiguously the presence of the endosymbionts together with the specific probe inside the host cells.

### Sequences comparison and phylogenetic analysis

The three molecular markers (18S rDNA, COI, ITS1-5.8S-ITS2) employed to characterize *P*. *chlorelligerum* were compared with sequences already present in database using BLASTN. In order to verify previous results and clarify uncertain sequences, cells from the German population (kindly provided by Dr. S. Krenek) were used to resequence all three molecular markers.

All phylogenetic analyses were performed both with Maximum Likelihood (ML) and Bayesian Inference (BI) methods. In case of ML, trees were calculated with 1000 bootstrap pseudoreplicates using PHYML software 3.0 [50] from ARB package [51]. BI was carried out with MrBayes 3.2 [52] running three runs, with one cold and three heated Monte Carlo Markov chains each, for 1000000 generations with a burn-in of 25%. After verifying that the average standard deviation of the split frequencies had reached a value 0.01 or below, the runs were stopped.

The optimal model of nucleotide substitution was chosen according to the Akaike Information Criterion (AIC) calculated with jModelTest [53], which selected the GTR + I + G model (using a discrete, four-categories gamma function) in all.

The 18S rRNA gene sequences were firstly aligned against more than 3500 ciliates sequences from SILVA 119.1 database [46] with ARB software package, then the alignment was optimized manually according to 18S rDNA secondary structure. Phylogenetic analysis was inferred using 34 sequences of ciliates belonging to the order *Peniculida* and the final alignment consisted of 1646 nucleotides (S1 Alignment).

The ITS1-5.8S-ITS2 secondary structures were predicted using mFOLD version 3.2 [54], and the presence of compensating base changes (CBCs) was examined as shown in Coleman [55]. After all, in total 29 sequences were aligned with MUSCLE 3.8.31 [56], and phylogeny was inferred with a final alignment of 999 characters (S2 Alignment).

The obtained 16S rRNA gene sequences of the novel bacterial endosymbionts were firstly aligned against those present in the latest version of SILVA database (release 123) using the ARB software package. Afterwards, the alignment was manually edited in order to optimize base-pairing in the predicted rRNA stem regions. The aligned sequences were reduced at both ends to the length of the shortest one, and gaps were treated as a missing character.

Phylogenetic analysis was performed using 44 sequences from the order *Rickettsiales* and 6 alphaproteobacterial sequences as out-group. The final alignment contained 1356 nucleotide columns, which were used to infer phylogeny (S3 Alignment).

## Results

### Diversity of ciliates in Peterhof ditch

Ciliates were repeatedly collected in a freshwater ditch (Peterhof, English park) during all seasons for one year. Throughout sampling, the temperature of water varied from +1°C (January) to +23°C (August), and pH ranged from 6.6 to 7.15 (measured in spring, summer, and autumn). In the same samples, taken from the water column and from the upper layer of bottom, numerous different ciliates with algal cytoplasmic symbionts were detected: *Frontonia vernalis*, *Frontonia leucas*, *Stentor polymorphus*, *Climacostomum virens*, *Spirostomum semivirescens*, *Loxodes rostrum*, *Pelagotrix plancticola*, *Microthorax viridis*, *Spathidium chlorelligerum*, *Prorodon niveus*, *Euplotes daidaleos*, *Stichotricha secunda*, *Dileptus* sp., and *Paramecium bursaria*. Four other morphospecies of *Paramecium* genus were found in the water body: *P*. *caudatum*, *P*. *aurelia*, *P*. *multimicronucleatum*, and *P*. *putrinum*. Bacteria belonging to *Holospora* genus, namely *H*. *acuminata* and *H*. *obtusa*, were retrieved in cells of *P*. *bursaria* and *P*. *caudatum* respectively.

The number of *P*. *chlorelligerum* cells present in different samples, which were collected in a distance of about 30–50 cm from each other, might vary significantly, but these ciliates were always present. *P*. *chlorelligerum* was also omnipresent through all the year seasons. In December–February all surface of the ditch was frozen, hence in January 2015 the surface was covered with ice 10 cm thick (Fig 1B). Despite this, the general composition of ciliate community under the ice was relatively similar to the one observed during the other seasons, but with much less abundance.

In absence of a special cultivation conditions (see Materials and Methods) *P*. *chlorelligerum* usually disappeared in few days after collection from the original samples, instead, for instance, *Spirostomum semivirescens* or *Frontonia vernalis* could survive in samples at least several weeks, while *Loxodes rostrum* was present for more than a month.

### Biology of *Paramecium chlorelligerum*

#### Ciliate morphology and ultrastructure

**Fig 2:**
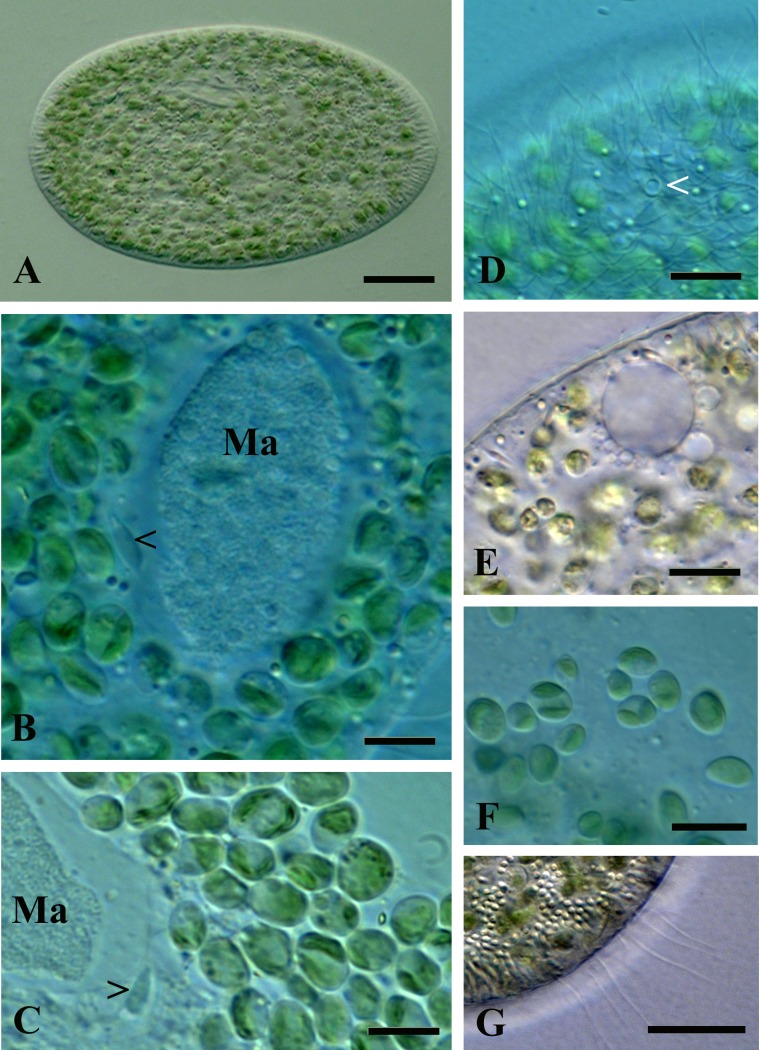
Morphological features of *P*. *chlorelligerum*. (A) General view of living cell; (B) nuclear apparatus: macronucleus (Ma) and micronucleus (black arrowhead) are indicated; (C) squashed cell: macronucleus (Ma) and micronucleus (black arrowhead) are indicated; (D) pore of contractile vacuole (white arrowhead); (E) contractile vacuole with satellite vesicles; (F) cytoplasmic symbiotic algae released from the squashed ciliate cell; (G) tuft of long caudal cilia. Scale bars: 17 μm (A); 6 μm (B, C); 10 μm (D-F); 20 μm (G).

**Fig 3:**
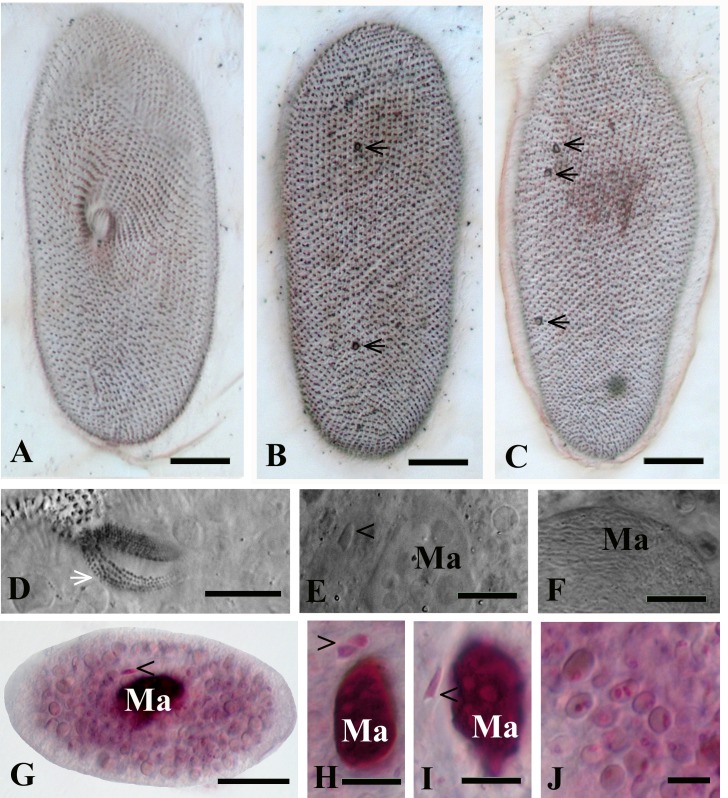
**Silver nitrate impregnation (A-C) and Feulgen staining (G-J) of *P*. *chlorelligerum*.** (A) ventral and (B, C) dorsal sides of the ciliate; position of oral aperture and pores of contractile vacuoles (black arrows) well visible; (D) oral ciliature: two peniculi and quadrulus (white arrow); (E) nuclear apparatus: macronucleus (Ma) and micronucleus (black arrowhead) are indicated; (F) macronucleus (Ma) infected with bacteria; (G) general view of a ciliate after Feulgen staining; nuclear apparatus with two (H) and one (I) micronuclei; (J) symbiotic algae in the cytoplasm of ciliate. Scale bars: 13 μm (A-C); 20 μm (D, G); 8 μm (E, F); 10 μm (H, I); 7 μm (J).

The cell morphology of *P*. *chlorelligerum* from the newly discovered population matches quite well with the description presented in two previous publications [11, 12]. Cell shape was mostly ellipsoidal, and the average size of living specimen was 113 x 45 μm; while impregnated ciliates were a bit smaller 106 x 44 μm (Figs 2A and 3). The main morphological features which allowed to discriminate the species were: 1- two contractile vacuoles (CV) of “vesicle” type (without collecting canals) terminated in cortex by single pore (CVP) each in 85% (n = 20) of population, while a small fraction of cells presented two pores in one of the two CV (Figs 2D, 2E, 3B and 3C); 2- caudal cilia (about 10), which were twice longer than the rest of somatic ciliature (about 10 μm (Fig 2G)); 3- relatively small (4.5–6.0 x 2.0–2.5μm) single spindle-shaped micronucleus of “compact” type with a hyaline “achromatic cap” (only once 2 micronuclei were detected in a cell) (Figs 2B, 2C, 3E, 3G and 3I). Just after cell division and in case of bimicronuclear cell, the form of the micronucleus was different from spindle-shape (Fig 3H). In the cells of investigated clones, oral opening was located slightly in front of cell equator line; oral ciliature manifested sometimes irregularity (Fig 3A and 3E) of quadrulus (4 or 5 rows), but the classical composition (4 rows) was the most common.

**Fig 4:**
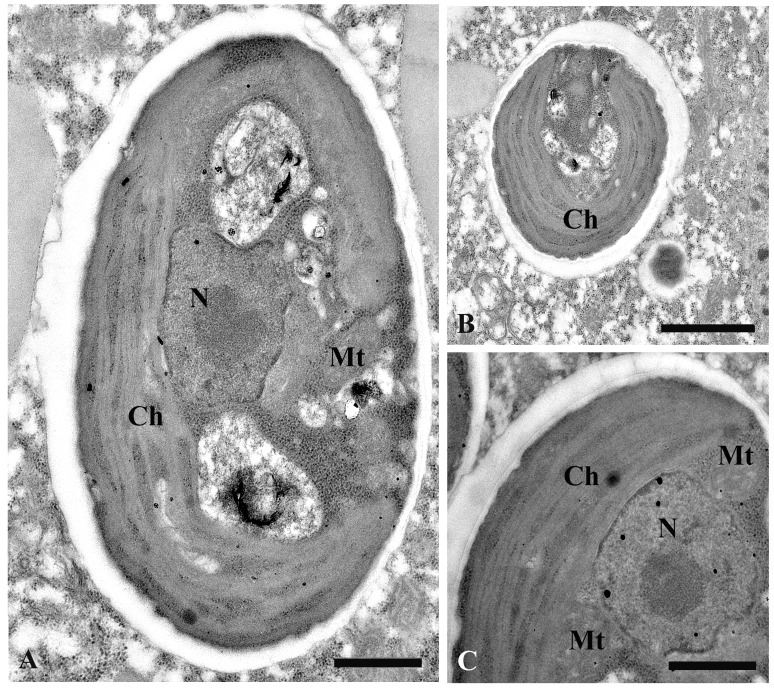
Ultrastructure of cytoplasmic symbiotic algae. (A) longitudinal section of alga cell; (B) cross section of alga cell; (C) part of the cell with nucleus. Nucleus (N), chloroplast (Ch) and mitochondria (Mt) are indicated. Scale bars: 0.5 μm (A), 0.4 μm (C); 3 μm (B).

The cytoplasm of *P*. *chlorelligerum* always contained a number (several hundreds) of green algae, which reminded at a first glance *Chlorella* sp. Slightly ellipsoidal algae cells were 5–7 x 3–6 μm in size. These endosymbionts were located in perialgal vacuoles, and were characterized by one cap-like chloroplast without distinctive pyrenoid inside. A single nucleus was generally situated close to chloroplast, but in some algae cells the nucleus was doubled. When the nucleus was stained by Feulgen, it looked as a vesicle structure (because of big nucleolus) localized in periphery of the cell (Figs 2A–2C, 2F, 3J and 4).

#### Endosymbiotic algae identification and cultivation attempts

Endosymbiotic algae were identified molecularly by PCR on fixed cells of *P*. *chlorelligerum*. A partial (1223 bp long, GenBank KX669637) 18S rRNA gene sequence was obtained, and its identity was 99.3% with *Meyerella planctonica* isolated from environmental samples [57, 58] (Table 2). When this sequence was compared to the one of German *P*. *chlorelligerum* endosymbiotic algae (GenBank JX010741), identity was 98.9%, thus confirming the presence of *Meyerella* sp. as endosymbiont of this green ciliate.

**Table 2:** 18S rDNA gene sequences identities among members of *Meyerella* genus.

	Meyerella planctonica isolate from Lake Itaska (AY543042)	Meyerella sp. isolate from Utah desert (KF693808)	Endosymbiont of German P. chlorelligerum (JX010741)	Endosymbiont of Russian P. chlorelligerum (KX669637)
Meyerella planctonica isolate from Lake Itaska (AY543042)	/	98.27	99.39	99.34
Meyerella sp. isolate from Utah desert (KF693808)		/	97.90	97.58
Endosymbiont of German P. chlorelligerum (JX010741)			/	98.90
Endosymbiont of Russian P. chlorelligerum (KX669637)				/

**Fig 5:**
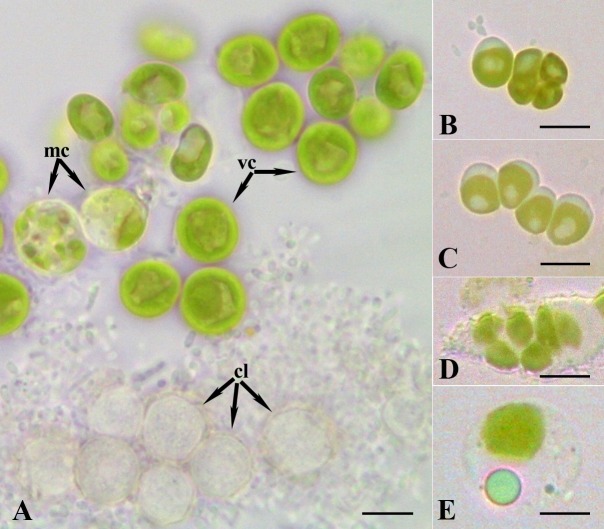
Diversity of cells in the culture of presumably ex-symbiotic algae obtained from *P*. *chlorelligerum*. (A) The young vegetative cells (vc), the mature cells (mc), and the cyst-like cells (cl); (B) division into four cells as tetrads or (C) chains; (D) production of autospores; (E) mature cell with a lipid droplet in cytoplasm. Scale bars: 7 μm (A); 5 μm (B-E).

All algae cultures obtained directly from the initially grown on BBM plates were clearly not morphologically resembling the algae inhabiting the *P*. *chlorelligerum* cytoplasm, as they had distinct pyrenoid (data not shown). By partial 18S rDNA sequencing they were identified as *Chlorella sorokiniana*. Algae present in the single culture started from cyst-like cells (see Material and Methods) were morphologically similar to symbiotic algae observed in *P*. *chlorelligerum* cytoplasmic symbionts (Fig 5A). These opaque green algae lacked of pyrenoid. Several life stages were observed (Fig 5A): young vegetative cells, mature cells actively producing and secreting lipids (Fig 5E), a typical characteristic for *Meyerella* growing in restrictive conditions [59], and cyst-like cells. These algae multiplied either by dividing into four cells as tetrads or chains (Fig 5B and 5C), or producing autospores (Fig 5D), another distinctive feature of free-living *M*. *planctonica* [58]. Mature cells formed double-layered shells, thus, probably, preparing for encystment. Formation of cyst-like cells is typical for free-living algae which have to survive in unfavorable conditions, such as desiccation [60]. Such cyst-like cells are also known as hypnospores [61] and they are characterized by thick multilayered cell walls, and by accumulation of lipids, starch, and phenolic substances [60]. Unfortunately, the culture of these algae suddenly stopped growing in liquid BBM within a week, and all cells died before reaching sufficient amount for DNA extraction. Attempt to transfer them back from liquid medium to BBM agar plates also failed.

#### Molecular characterization, sequences comparison and phylogenetic analysis of *Paramecium chlorelligerum*

The almost complete 18S rRNA gene sequences (1716 bp) were obtained by direct sequencing after semi-nested amplification from the two strains (HSG1-11 GenBank KX669629, and HSG2-12 GenBank KX669630), and they were identical. These “Russian” *P*. *chlorelligerum* sequences were 99.8% identical to representatives of the “German” population [1, 12]. In those sequences, respectively three (position 1029, 1298 and 1310) and two (positions 1298 and 1310) mismatches were present. The two 18S rDNA sequences produced from German population [1, 12] were differing in a nucleotide, a W in [12]. To resolve this ambiguity, we resequenced a sample derived from the German population obtaining the same result as [1]. Thus, we can confidently assume that 18S rDNA from German population is differing from Russian one in two nucleotides. Intriguingly these two mutations are located on opposite site of the stem with the second mutation re-establishing the correct secondary structure (they represent a compensatory base changes (CBC)); the two mutations are consequently not evolutionary independent and possibly highlight a highly variable stem in *P*. *chlorelligerum*.

The two Russian strains had an identical sequence of COI gene (760 bp, HSG1-11 GenBank KX669632, HSG2-12 GenBank KX669633), which was 100% homologous to COI gene of the ciliates from German population [1]. On the contrary, the other COI sequence attributed in GenBank (KF110720) to *P*. *chlorelligerum* appeared to be completely different and probably is the result of organism misidentification. Indeed, in recent COI phylogenetic reconstruction [1], this sequence is present in the outgroup composed by different ciliate species.

ITS1-5.8S-ITS2 sequences were 1080 bp long and 100% identical among all *P*. *chlorelligerum* characterized by us (HSG1-11 GenBank KX669629, HSG2-12 GenBank KX669630 and German population GenBank KX669631), and no CBC were found. Again, the GenBank sequence KF110708 attributed to *P*. *chlorelligerum* seems to be the result of a mixture of sequences belonging to *Paramecium* and some other ciliate, probably prostomatean (e.g. *Prorodon* sp.), thus showing a misidentification of ciliate cells or contamination during DNA sample preparation.

**Fig 6:**
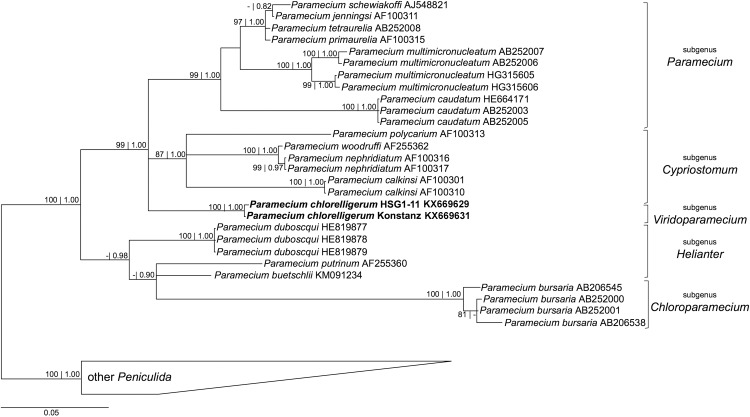
Bayesian inference phylogenetic tree of genus *Paramecium* based on 18S rDNA gene sequences. Numbers associated to each node represent bootstraps values inferred after 1000 pseudoreplicates and Bayesian poster probabilities (values below 70 | 0.70 are not shown). Sequences in bold were characterized in this study. The bar stands for an estimated genetic distance of 0.05.

**Fig 7:**
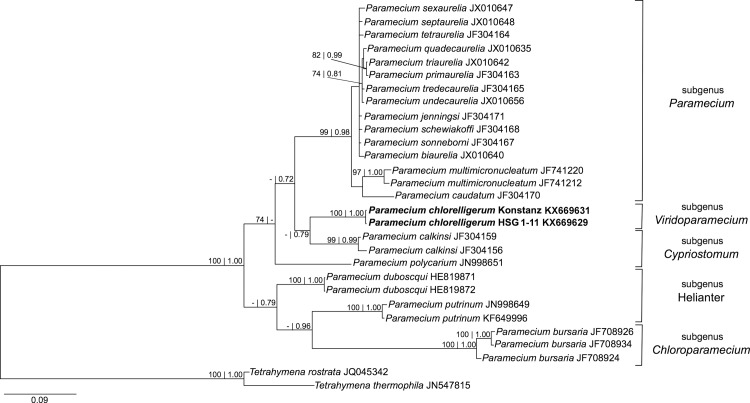
Bayesian inference phylogenetic tree of genus *Paramecium* based on ITS1-5.8S-ITS2 sequences. Numbers associated to each node represent bootstraps values inferred after 1000 pseudoreplicates and Bayesian poster probabilities (values below 70 | 0.70 are not shown). Sequences in bold were characterized in this study. The bar stands for an estimated genetic distance of 0.09.

After the model GTR + I + G was chosen by jModelTest, phylogeny for 18S rDNA and ITS1-5.8S-ITS2 were inferred. As previously shown [1, 12], the 18S rRNA gene phylogeny confirmed the position of *P*. *chlorelligerum* as clustering with the subgenera *Cypriostomum* and *Paramecium* (Fig 6). These results were confirmed also by ITS1-5.8S-ITS2 gene phylogeny, in which *P*. *chlorelligerum* was slightly associated as sister group of all species of the subgenus *Cypriostomum* as shown in Fig 7. The faint differences between German and Russian *P*. *chlorelligerum* 18S rDNA sequences are visible also on the tree (Fig 6), which is in agreement with the last 18S rDNA phylogenetic tree published on *Paramecium* genus [1]. On the contrary, our ITS1-5.8S-ITS2 phylogenetic tree (Fig 7) presents a different situation. First of all, we provided new and correct sequences of *P*. *chlorelligerum* and its position is completely different from previous studies. Indeed, this green ciliate clusters as sister group with *Cypriostomum* subgenus, which is in agreement with other phylogenetic reconstructions based on other molecular markers (e.g. 18S rDNA, COI) [1]. On the other hand, ITS1-5.8S-ITS2 phylogeny shows that *P*. *polycarium* does not cluster with the other members of *Cypriostomum* group, thus enlightening a necessity of revising this subgenus from a morphological and molecular point of view; and some positions within *Paramecium aurelia* complex are not solved due to a probably lack of sequences.

### Bacterial morphology and its life cycle

In the sampled *P*. *chlorelligerum* populations, 5–7% of cells manifested macronuclear infection by bacteria morphologically resembling *Holospora* sp. After subcloning from two samples in 2014 and 2015, two infected strains were established (HSG1-11, HSG2-12), and cells of these were investigated from morphological and molecular points of view.

**Fig 8:**
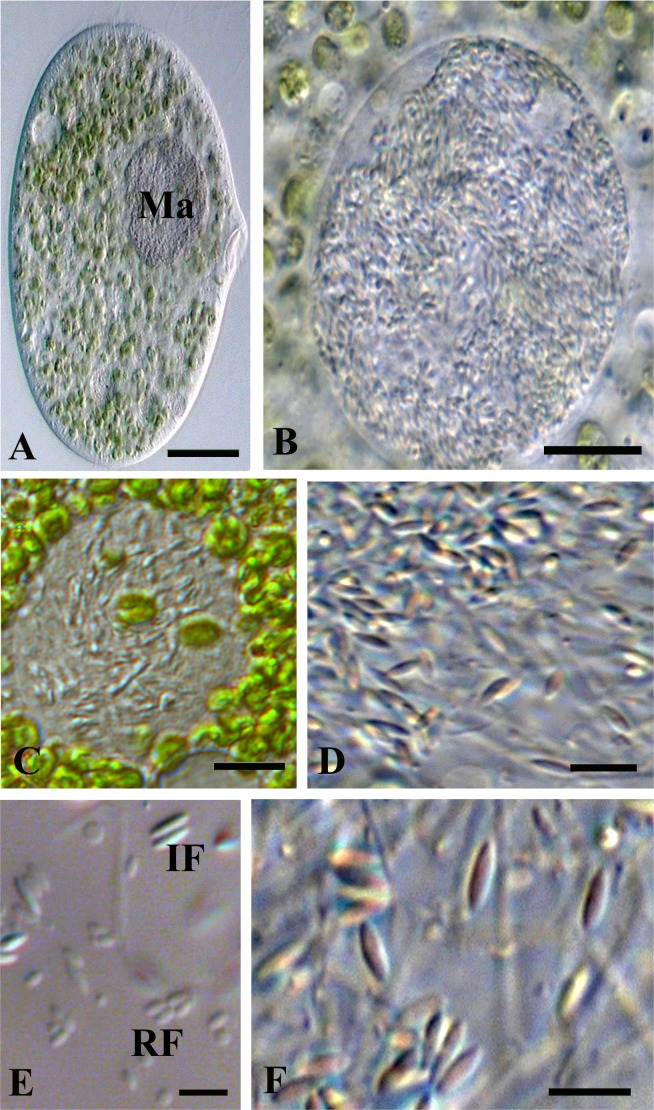
*P*. *chlorelligerum* infected with *Holospora*-like bacterium. (A) general view of infected cell: macronucleus (Ma) is indicated; (B) highly infected macronucleus; (C) slightly infected macronucleus; (D) bacteria releasing from the squashed macronucleus; (E) infectious (IF) and reproductive (RF) forms of the bacterium; (F) spindle-like IFs under high magnification. Scale bars: 13 μm (A); 10 μm (B); 8 μm (C); 5 μm (E, F).

**Fig 9:**
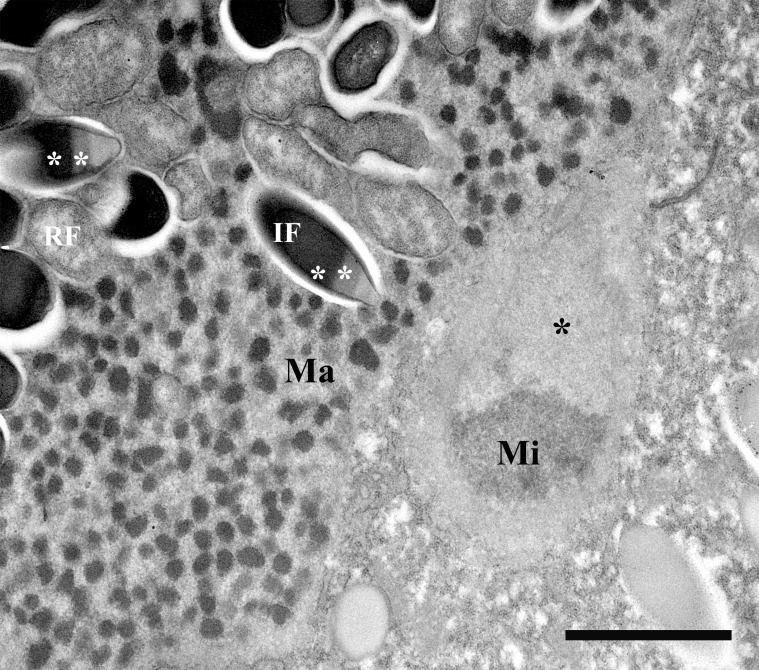
Micrograph of *P*. *chlorelligerum* macronucleus infected with *Holospora*-like bacterium. In karyoplasm both infectious (IF) and reproductive (RF) forms of the bacterium are visible. IF manifest double composition of periplasm (white asterisks). Attached to the macronucleus (Ma) a part of the micronucleus (Mi) with distinctive a hyaline “achromatic cap” (black asterisk). Scale bar: 2 μm.

**Fig 10:**
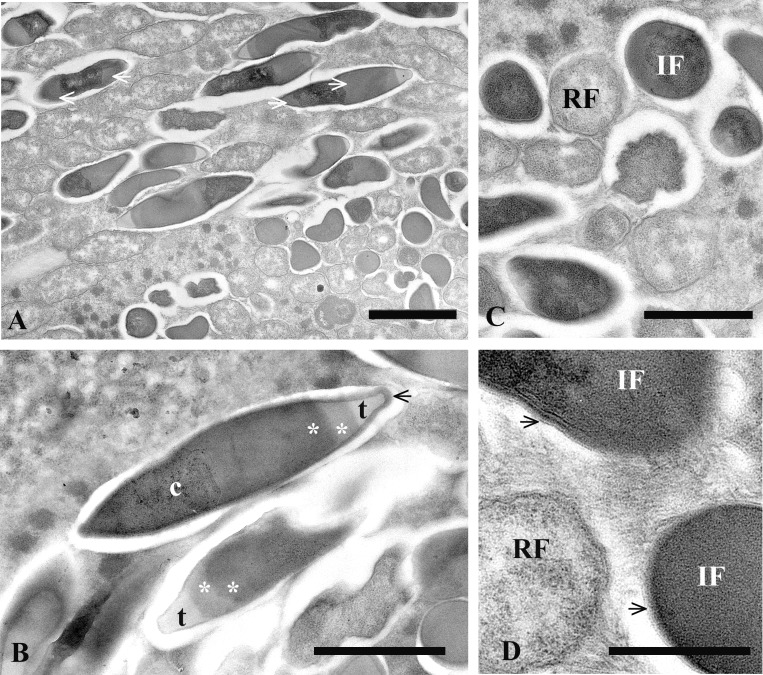
Peculiarities of *Holospora*-like bacterium. (A) part of infected macronucleus; (B) composition of infectious form: cytoplasm (c), periplasm regions with different density (white asterisks) and recognition tip (t); (C) cross sections of infectious (IF) and reproductive (RF) forms; (D) fine fibrous material can be presented on the surface of some of IFs (black arrows). Scale bars: 2 μm (A); 1.5 μm (B); 1 μm (C); 0.6 μm (D).

Two types of straight non-motile bacteria with different size and structure were observed in the infected macronucleus. There were two morphological forms, which looked like infectious (IF) and reproductive (RF) forms of classical *Holospora*-like bacteria [13]. RFs were short, slightly ovoid, and their length was 1–3 μm; IFs were spindle-shaped and 3–5 μm long; diameter of both forms of the bacterium was estimated as 0.6–0.7 μm (Figs 8–10). The RF manifested typical homogenous prokaryotic cytoplasm (Figs 9, 10A, 10B and 10D). The bacterium IF with distinctive tapered ends displayed differentiated cytoplasmic and periplasmic parts and a recognition tip-like structure (Fig 10A and 10B). The recognition tip contained less osmiophilic material and reminded those of other *Holospora* species, but periplasmic region always showed two different parts, a larger and denser one, and another one more transparent. The latter was located close to recognition tip structure and looked as a minor part of periplasm (Figs 9, 10A and 10B). Sometimes the same cell could manifest two strips of dense periplasm, but the transparent part of it was always single and situated close to the recognition tip (Fig 10A and 10B). Another difference between IF and RF was the composition of its surface membrane. Indeed, in majority of IFs their surface was decorated with fine fibrous material (Fig 10B–10D).

The microorganism had a typical *Holospora*-like life cycle with alternation of IFs and RFs [13], and it could be completed (during experimental infection) only in 6 weeks, while the life cycle of *Holospora* sp. takes normally just 7–12 days [13]. In fact, the first IFs were detected in experimentally infected culture after 40 days. Before the nucleoplasm was populated exclusively by RFs. Sometimes, infected macronucleus might be overpopulated by symbionts with several hundred of IFs (Fig 8A–8C), becoming distinctively roundish and larger in size than uninfected ovoid nucleus (Figs 2B, 2C and 3F). During cultivation, growth of infected cells was as slow as a growth rate of uninfected ones, only 1–2 divisions per week.

The fate of infected cell lines was different. HSG1-11 manifested 100% infection, but after 6 months died. Instead, cells of HSG2-12 showed 100% of infection and then almost lost infection after 2 months of cultivation, but after 6 other months the strain recovered bacterium presence up to 10–90% (subpopulations of the same stock in different Petri dishes). It was possible to check only few dividers, but the connecting piece was never observed during division of infected macronucleus. The connecting piece, an equatorial part of the dividing host nucleus where the majority of IFs are gathered, is a feature manifested by all “classical” holosporas [62]. The daughter cells of infected *P*. *chlorelligerum* always inherited both bacterial forms in their macronuclei, and sometimes IFs could even produce a kind of clusters in the karyoplasm. The process of IFs release from the macronucleus into the ciliate cytoplasm was not investigated, but some bacteria were detected in cytoplasm during electron microscopy investigation.

### Molecular characterization and phylogenetic analysis of the bacterial endosymbiont

Almost complete 16S rRNA gene sequence (1404 bp) was obtained by direct sequencing from the two strains (HSG1-11 GenBank KX669635, and HSG2-12 GenBank KX669636) harboring endosymbionts. These were identical between each other and presented an identity value ranging from 96.5% to 98.1 with other *Holospora* species (Table 3).

**Table 3:** 16S rDNA gene sequences identities among members of *Holospora* genus and within the same species, when more than one sequence is present.

	*H*. *obtusa*	*H*. *undulata*	*H*.*elegans*	*H*. *curviuscula*	*H*. *acuminata*	“*Ca*. H. parva”
*H*. *obtusa*	99.59	98.15	98.08	96.29	96.47	96.55
*H*. *undulata*		/	99.92	97.29	97.30	97.64
*H*. *elegans*			/	97.02	96.76	97.22
*H*. *curviuscula*				99.71	97.90	97.22
*H*. *acuminata*					100.00	97.48
“*Ca*. H. parva”						100.00

**Fig 11:**
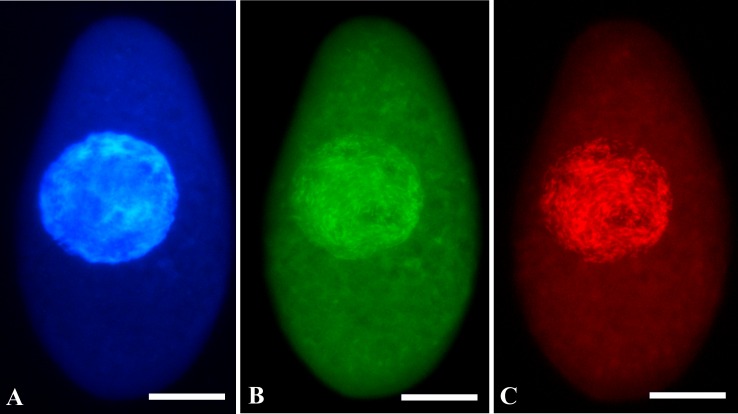
Fluorescence *in situ* hybridization results on fixed *P*. *chlorelligerum* cell infected with *Holospora*-like bacterium. (A) The ciliate macronucleus stained by DAPI; bacteria in the macronucleus are visualized both with eubacterial probe EUB338 (B) labeled with AlexaFluor® (green signal) and with the probe specific for *Holospora*-like bacterium HoloParv_645 labeled with Cy3 (red signal) (C). Scale bars: 20 μm.

A species-specific probe was designed and FISH experiments were carried out in order to detect the presence of the bacterial endosymbiont (Fig 11), as expected by “full-cycle rRNA approach” [49]. According to the 16S rRNA gene secondary structure and diversity of the *Holospora* genus sequences, regions suitable for species-specific probe design were limited. Indeed, the HoloParv_645 probe was designed in the only region, which resulted both accessible and also sufficiently variable. According to RDP results, HoloParv_645 probe may recognize, in addition to “*Ca*. Holospora parva”, a handful of sequences of uncultivable organisms. When a single mismatch was allowed, the number of hits significantly increased including also *H*. *obtusa*. In order to verify species-specificity of newly designed probe in condition of one mismatch, hybridization experiments with probe HoloParv_645 and *Paramecium caudatum* bearing *H*. *obtusa* were performed in 0, 15, 30% formamide conditions. The probe was never binding to *H*. *obtusa* at any formamide concentration (data not shown).

All cells screened showed the presence of bacteria in the host macronucleus (Fig 11). Moreover, signals from both probes EUB338 and HoloParv_645 overlapped, thus excluding the presence of other bacterial endosymbionts.

**Fig 12:**
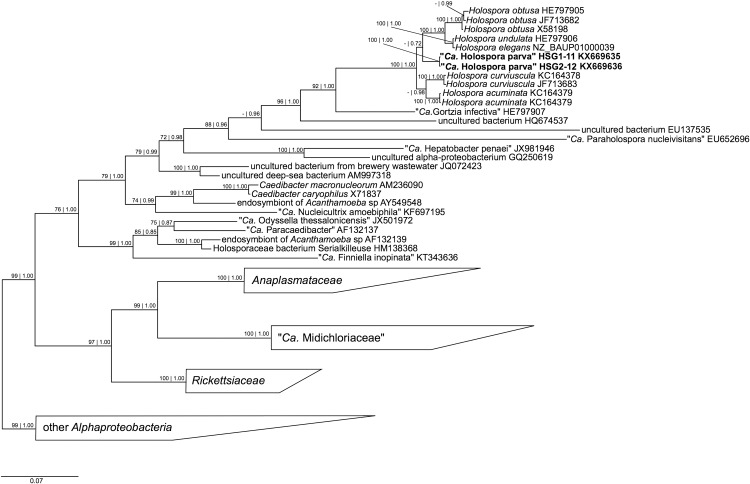
Bayesian inference phylogenetic tree of order *Rickettsiales* based on 16S rDNA gene sequences. Numbers associated to each node represent bootstraps values inferred after 1000 pseudoreplicates and Bayesian poster probabilities (values below 70 | 0.70 are not shown). Sequences in bold were characterized in this study. The bar stands for an estimated genetic distance of 0.07. “*Ca*.” stands for “*Candidatus*”.

ML and BI trees were inferred (Fig 12) after the GTR+I+G substitution model was chosen by jModelTest. The ML and BI trees showed good statistical values of bootstrap and posterior probability in the majority of nodes. As shown in the Fig 12, the monophyly of the families within the order *Rickettsiales* was confirmed, and their evolutionary relations were in agreement with recent literature [63–67], namely *Rickettsiaceae* as sister group of *Anaplasmataceae*-“*Candidatus* Midichloriaceae” clade (99% bootstrap value for ML and 1.00 posterior probability for BI). All members of the *Holospora* genus cluster together forming a monophyletic group. The newly characterized “*Ca*. Holospora parva” branches independently as sister of the clade *H*. *obtusa + H*. *undulata + H*. *elegans*.

## Discussion

### The water body in Peterhof, Russia, as a diversity hot-spot for green ciliated protists

The newly found freshwater locality manifested very unusual high biodiversity of green ciliates, at least fifteen species. In spite of 40 years practices in ciliate sampling in Russia, as well as in different parts of the World [68], we never found so high diversity of such ciliates in the same place. Some of mentioned species (e.g. *Loxodes rostrum*, *Pelagotrix plancticola*, *Microthorax viridis*, *Spathidium chlorelligerum*, *Prorodon niveus*, *Frontonia vernalis* and *Stichotricha secunda*), were never previously recorded in Russia. In literature, the case of six green ciliate species present in the same pond was treated as “spectacular abundance” [69] and reported number of ciliate species associated with endosymbiotic algae could be from one to eight in the same water body [70–72]. Indeed, the finding of so many green ciliate species demonstrates that Peterhof ditch can be considered as a hot-spot for ciliates inhabited by green algae. To our best knowledge, the diversity of ciliates with cytoplasmic green algae symbionts was higher only in Simmelried moorland ponds, nearby the town of Konstanz, where 30 species were found [73]. These two localities hosting *P*. *chlorelligerum* shared some general ecological features. On one hand, ground flora is rather different between Simmelried and Peterhof biotopes. Moss (*Sphagnum fallax*) which is a dominant plant in Simmelried, does not grow in proximity to the Peterhof ditch. Also edificatory trees in Simmelried were *Pinus sylvestris*, *Pinus mungo*, *Picea abies*, and *Betula* sp. with, probably, *Alnus* sp. (according to pictures from [73]), while in Peterhof the most common trees close to the ditch were *Betula pendula*, *Alnus glutinosa*, *Salix* sp., and *Quercus robur* (minority). However, at the same time both areas are humid and a bit marshy, and the waterbodies possess similar set of water vegetation: *Lemna* sp., *Carex* sp., *Phragmites australis*, *Typha* sp. and *Caricetum* sp. (minority in Peterhof). Some water plants like *Cladium marisci* absent in Peterhof just indicate more south localization of Simmelried. This similarity between Simmelried and Peterhof water bodies gives ground to doubt another finding of *P*. *chlorelligerum* in Romania, Herăstrău Lake, Bucharest [74] as that lake is big, rather clean and does not remind at all, according to the water vegetation set, the first two water bodies. On the contrary, the first description made by A. Kahl [11], even if short, indicates mire environment: “I know it only from the mud of a mire puddle” (page 830). So, we can assume that rather particular environmental conditions are needed for *P*. *chlorelligerum* presence. Probably specific quantities of microelements and substances present in water and soil of these marshy biotopes produce favorable conditions for specific water vegetation and stimulate the growth of unicellular algae prone for symbiosis with protists [75]. Such mire environments are not yet so much studied; if our considerations are correct, we can predict to find *P*. *chlorelligerum* and diverse green ciliates in some similar biotopes.

### Biology of *Paramecium chlorelligerum*

#### General morphology

Morphological characteristics of *P*. *chlorelligerum* from Russia match quite well with original description and redescription [11, 12]. Some small variations occurred in cell size, position of oral aperture, composition of the quadrulus and the number of contractile vacuole pores. Small morphometric deviations could be explained, probably, by measurement technique; on the contrary, quadrulus structure and variable number of pores could be explained by different geographical origin of the two investigated populations. General structure of contractile vacuole and single spindle-shaped micronucleus of “compact” type with a hyaline “achromatic cap” are stable for representatives of *P*. *chlorelligerum* and allow to identify it unequivocally, and, in particular, to discriminate between *P*. *chlorelligerum* and another green *Paramecium*, *P*. *bursaria*. However, it is worth mentioning after the German colleagues that *P*. *chlorelligerum* might have been overlooked many times in natural samples, as at a glance check it is very easy to confuse with *P*. *bursaria* or even with green prostomateans [12]. Thus, the frequency of *P*. *chlorelligerum* in nature might be significantly underestimated.

#### Sequence comparison, molecular variability and phylogeny of *Paramecium chlorelligerum*

The retrieval of another *P*. *chlorelligerum* population in a new location gave the possibility to investigate in more detail this rare green ciliate both from molecular and phylogenetic points of view. For this purpose, three molecular markers (18S rDNA, COI, ITS1-5.8S-ITS2) with different rate of evolution were employed (Figs 6 and 7).

The novel Russian locality hosted a very stable and homogeneous population from genetic point of view, and, thus, probably, isolated population as no difference was observed in the diverse molecular markers used to characterize strains isolated in different years. Our recharacterization of the German population confirmed previous data by Krenek et al. [1] and provided a novel and correct ITS1-5.8S-ITS2 sequence for this ciliate. Our phylogenetic reconstructions supported the previous ones inferred on 18S rDNA and COI [1, 12] and provided a novel phylogenetic tree based on ITS1-5.8S-ITS2, which confirmed the position of this rare ciliate species as a sister group of *Cypriostomum* subgenus, although with a low support. Unfortunately, it was not possible to infer phylogeny on the complete rDNA locus, as for most of *Paramecium* species present in databases some genes are missing. The comparison of *P*. *chlorelligerum* sequences revealed that this species displays a very low genetic variability, even in geographically separated populations. Indeed, only one out of the three molecular markers showed differences between the German and the Russian *P*. *chlorelligerum* populations. COI and ITS1-5.8S-ITS2, molecular markers usually employed to investigate intraspecific diversity, did not show any difference in the populations as they were 100% identical. Surprisingly, evolutionary more conserved 18S rDNA presented a couple of mismatches (CBC), which could be suitable to design a population-specific FISH probe, as an alternative and faster method to discriminate strains within the same morphospecies [76, 77]. The same phenomenon, but less emphasized, was found also in other members of the genus *Paramecium* [46, 78], where intraspecific variability was higher in 18S rDNA sequence than in the usually less conserved markers.

Nevertheless, the increase of sampling and studies on marshy environments could lead to the retrieval of other *P*. *chlorelligerum* populations, thus allowing a deeper understanding of the real genetic diversity of this rare ciliate. Furthermore, as several sequences in GenBank have been incorrectly assigned to *P*. *chlorelligerum* (KF110720, KF110708), now it becomes more and more important to combine molecular tools with morphological analysis of the studied microorganism to avoid further mistakes.

#### Characterization and ecology of endosymbiotic algae

The characterization of Russian *P*. *chlorelligerum* algal endosymbionts was performed both morphologically and molecularly, and they were identified as members of the *Meyerella* genus. This algal genus splits off the *Chlorella* group basally [57]. The main discriminating morphological feature of this genus is absence of pyrenoid in chloroplasts. This feature characterizes only three groups of green microalgae, namely *Kalinella*, *Leptochlorella* and *Meyerella* [79, 80]. The presence of a single chloroplast was revealed in symbiotic algae of Russian *P*. *chlorelligerum* (Fig 4), thus resembling *M*. *planktonica* description [58]. Still, ultrastucture of *M*. *planktonica* is not identical with symbionts from *P*. *chlorelligerum*. Some morphological differences were revealed also between *Meyerella* sp. from Russian paramecia and other members of the *Meyerella* genus. At the moment two free-living species of *Meyerella* have been described, namely *M*. *planktonica* from the Itaska lake in Minnesota [58], and *Meyerella* sp. from the soil crust in Utah desert area [57]; also microalgae inhabiting German *P*. *chlorelligerum* have been identified as *Meyerella* sp. [12]. In particular, two chromatophores, leaving blank one polar region, were detected in symbiotic algae of German *P*. *chlorelligerum* [12], while *Meyerella* sp. isolated from the soil crusts was characterized by lobed or even fragmented chloroplasts [57]. Significant diversity within the *Meyerella* genus was observed also from molecular point of view. Indeed, two free-living *Meyerella* sp. were molecularly characterized [57, 58] and they displayed 98.3% similarity of 18S rDNA. At the same time, two 18S rDNA sequences of symbiotic *Meyerella* lineages were 98.9% identical. Comparison between free-living and symbiotic *Meyerella* sequences showed differences from 97.6 to 99.3% (Table 2). Thus, symbiotic *Meyerella* from Russian *P*. *chlorelligerum* was even more similar to *M*. *planctonica*, than two free-living species of *Meyerella* to each other.

*Meyerella* has never been reported as a symbiont of any other ciliate, except *P*. *chlorelligerum*. Until recently [79], all known symbioses between freshwater ciliates and algae generally have been presumed to involve representatives of *Chlorella* genus (Chlorophyta). Systematic revision of zoochlorellae [79], redescription of *P*. *chlorelligerum* [12], new data on green symbionts of *Loxodes rostrum* [71] and on *Tetrahymena utriculariae* [81], changed this view on algae inhabiting cytoplasm of ciliates. Comparative analysis of different *Chlorella*-like symbionts [79] revealed five phylogenetically distinct symbionts, namely *Chlorella*, *Choricystis*, *Coccomyxa*, *Scenedesmus* and *Micractinium*, which could be found in several ciliates. Recently representatives of a new green algae group called “Chlorb” (probably forming a new genus) were retrieved from four green ciliates isolated from Lake Biwa in Japan [82]. Now also the genus *Meyerella* should be added to the list of symbiosis-forming algae after its retrieval in the cytoplasm of both German [12] and Russian *P*. *chlorelligerum*. Two independent findings of these algae in geographically diverse populations of *P*. *chlorelligerum* allow to suggest that this symbiotic association is not occasional. The failure in stable cultivation of ex-symbiotic *Meyerella* on BBM, considered as a quality standard for maintenance of unicellular algae including ex-symbiotic *Chlorella* and *Micractinium* [83], may be explained by the lack of some special conditions which these algae meet inside the ciliate cell. For example, these endosymbiotic algae may get a certain advantage living in a host with slow growth rate, as propagation enhanced by artificially favorable conditions could be too fast. Other possible explanations of unsuccessful cultivation attempts of symbiotic *Meyerella* may include low frequency of cyst-like cells formation readily in *Paramecium*, occasional overgrowth of it by *Chlorella* if the latter are present in medium or engulfed but not digested by the ciliate, and even presence of a latent virus which activates and lyses the host cells when they start rapid growing outside of *Paramecium* [84, 85].

All these data indicate that there is a need of revision of *Meyerella* species, as this genus includes both free-living and diverse symbiotic algae which possess also morphological differences. Furthermore, our findings raise a question on the origin and evolution of symbiosis between these algae and ciliates and suggest that *P*. *chlorelligerum* from the two populations independently acquired *Meyerella* from the environment.

### Intracellular symbionts of *Paramecium chlorelligerum* are a novel *Holospora* species

The characterized bacterium shares many morphological and life cycle similarities with members of the *Holospora* genus. The pattern of features usually employed to identify bacteria of this genus was previously described for several species [86–94]. Indeed, alternation of IF and RF in their life cycle, high infectivity of the IF, nuclear specificity, and high selectivity for the host *Paramecium* species are considered to be distinctive features of *Holospora* species. The novel bacterium found in *P*. *chlorelligerum* macronucleus fits to all these criteria, and, thus, can be considered as a representative of the *Holospora* genus. This conclusion is also supported by ultrastructural data on morphology of IFs. The ultrastructure of IF slightly varies in the species of the *Holospora* genus. Among them, *P*. *chlorelligerum* symbiont is the smallest in size and according to shape and length reminds *H*. *acuminata* [87]. Finally, 16S rDNA sequence similarity with several *Holospora* species of circa 97% and phylogenetic analysis has shown that novel bacterial symbiont of *P*. *chlorelligerum* clusters with *H*. *obtusa*, *H*. *unduluta*, and *H*. *elegans*, thus unequivocally including it in the *Holospora* genus (Fig 12).

This new *Holospora* species proved to be infectious, as experiments in stock HSG2-12 showed that reinfection is a possible event in the *P*. *chlorelligerum* population. However, outcome of experimental infection was relatively low, and development of infection took much longer than in all other *Holospora* species. Low rate of this process is probably connected with physiology of the host ciliate, which is characterized by unusually slow reproduction and, consequently, may put certain limitations on the readiness of experimental (or native) infection. It is also possible that laboratory conditions are insufficient to allow faster growth of the host ciliate and, consequently, the symbiotic bacteria slow down their reproduction.

All members of the *Holospora* genus recover maximal support and form a monophyletic group (Fig 12). Up to now, the validity of the *Holosporaceae* family within the order *Rickettsiales* is under discussion. As shown in some recent literature based on 16S rDNA and genome phylogenies [23, 63, 64], the family *Holosporaceae* is placed as basal to the rest of *Rickettsiales* and within this order. Other studies utilizing different and less widespread molecular markers, such as 23S rDNA and protein-coding genes, placed *Holosporaceae* outside of the order *Rickettsiales* and suggested to rank it as a separate order [95–97]; a recent study formally elevated *Holosporaceae* to the order *Holosporales* providing an overall revision of families belonging to this new order [98].

However, our phylogenetic reconstruction of *Holosporaceae* and *Holosporales* is in accordance with previous studies [23, 63, 64, 98] and its inner relations are confirmed. Indeed, all *H*. *obtusa* sequences cluster together with *H*. *undulata* as sister group, and the earlier divergence of *Holospora* species associated with *P*. *bursaria* is confirmed and they cluster together [23]. Within the group of *P*. *caudatum* “classical” holosporas, the novel species from *P*. *chlorelligerum* occupies basal position, thus forming a separate lineage. “*Candidatus* Gortzia infectiva”, an *Holospora*-like bacterium, is confirmed as the closest relative of *Holospora* genus [23, 99].

These phylogenetic findings make doubtful the importance of inducing the connecting piece as another feature considered to be distinctive between two groups of holosporas. Connecting piece is the result of IF concentration in a particular median body during division process of the infected host nucleus facilitating further release of symbionts to environment [100]. It was shown that the presence of the connecting piece is characteristic for classical *Holospora* species (i.e. those infecting *P*. *caudatum* and *P*. *bursaria*), while this structure is not induced by other *Holospora*-like bacteria [13, 23, 62]. If present, this event could be considered as a particular adaptation of bacteria evolved to accomplish more efficiently the complex life-cycle, exploiting host cell divison machinery [100, 101]. This feature has not been found for two *Holospora* species infecting macronuclei of representatives of the *Frontonia* genus, namely *F*. *salmastra* and *F*. *leucas* [13, 68]. Moreover, the lack of inducing the connecting piece was observed also in “*Candidatus* Gortzia infectiva” from *P*. *jenningsi* [23]. Interestingly, the novel macronuclear endosymbiont of *P*. *chlorelligerum* also does not induce the connecting piece reminding “*Ca*. Gortzia infectiva” rather than “classical” holosporas, though phylogenetically it is closer to the latter. Considering both morphological and molecular data, we can not claim anymore the presence of the connecting piece as an apomorphic feature for all *Holospora* species.

According to the current microbiological rules [102] for nomenclature, we can coin only the status of “*Candidatus*” for the novel macronuclear bacterium of *P*. *chlorelligerum* as a representative of non-cultivable microorganisms.

### Description of “*Candidatus* Holospora parva” sp. nov.

*Holospora parva* (N.L. adj. parva, a little)

Rod-shaped gram-negative bacteria, with differentiated reproductive (RF) and infectious (IF) forms. RF 1.0–3.0×0.6–0.7 μm with homogeneous cytoplasm without visible inclusions.

IF 3.0–5.0×0.6–0.7 μm straight rods, with distinctive tapered ends, extensive periplasmic space divided into two parts with different osmiophilic density, and a recognition tip. Does not induce the formation of a distinctive connecting piece during host cell division. Macronuclear endosymbiont of the free-living ciliate *Paramecium chlorelligerum*, identified in samples taken from permanent ditch in English park of Peterhof (St. Petersburg district, Russia). Capable of horizontal and vertical transmission in the host species. Basis of assignment: 16S rRNA gene sequence (GenBank KX669636) and positive matching with the 16S rRNA-targeting oligonucleotide probe HoloParv_645; morphological characters pattern as above. Uncultured thus far.

## Supporting Information

S1 Alignment Alignment of 18S rDNA of *Paramecium* genus. 34 sequences of ciliates belonging to the order *Peniculida* were aligned to perform phylogenetical analysis of *Paramecium chlorelligerum*. The resulting 1646 nucleotides columns are presented here. (S1 Alignment)
(TXT)

S2 Alignment Alignment of ITS1-5.8S-ITS2 of *Paramecium* genus. 29 sequences of ciliates were aligned to perform phylogenetical analysis of *Paramecium chlorelligerum*. The resulting 999 nucleotides columns are presented here. (S2 Alignment)
(TXT)

S3 Alignment Alignment of 16S rDNA of *Rickettsiales* order. 44 sequences of the order *Rickettsiales* and 6 other alphaproteobacteria as outgroup were aligned to perform phylogenetical analysis of *Paramecium chlorelligerum* bacterial endosymbiont. The resulting 1356 nucleotides columns are presented here. (S3 Alignment)
(TXT)
